# 1-Benzyl-3-methyl-3′,5′-diphenyl­spiro­[quinoxaline-2(1*H*),2′(3′*H*)-1,3,4-thia­diazole]

**DOI:** 10.1107/S1600536811052731

**Published:** 2011-12-14

**Authors:** Caleb Ahoya Anothane, Rachid Bouhfid, El Mokhtar Essassi, Seik Weng Ng

**Affiliations:** a Laboratoire de Chimie Organique Hétérocyclique, Pôle de Compétences Pharmacochimie, Université Mohammed V-Agdal, BP 1014 Avenue Ibn Batout, Rabat, Morocco; b Institute of Nanomaterials and Nanotechnology, MAScIR, Avenue de l’Armée Royale, Rabat, Morocco; c Department of Chemistry, University of Malaya, 50603 Kuala Lumpur, Malaysia; d Chemistry Department, King Abdulaziz University, PO Box 80203 Jeddah, Saudi Arabia

## Abstract

In the title spiro compound, C_29_H_24_N_4_S, the quinoxaline and thia­diazole ring systems share a common C atom; their mean planes are aligned at 87.0 (1)° in one mol­ecule and at 84.1 (1)° in the other independent mol­ecule. The thia­zole ring possesses two aromatic ring substituents and is roughly coplanar with these rings [the dihedral angles between the thia­diazole and phenyl rings are 10.7 (1) and 11.7 (1)° in one mol­ecule, and 16.8 (1) and 17.7 (1)° in the other]. The aromatic ring of the benzyl unit of one mol­ecule is disordered over two orientations in a 1:1 ratio.

## Related literature

For the structure of a related mol­ecule, see: Anothane *et al.* (2010 ▶).
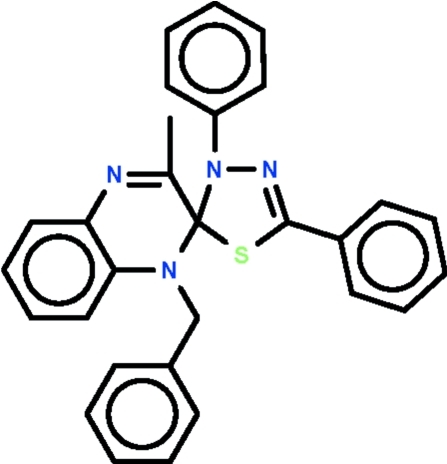


## Experimental

### 

#### Crystal data

C_29_H_24_N_4_S
*M* *_r_* = 460.58
Triclinic, 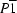

*a* = 13.5441 (2) Å
*b* = 14.8971 (2) Å
*c* = 15.0149 (2) Å
α = 66.431 (1)°
β = 63.921 (1)°
γ = 65.275 (1)°
*V* = 2383.45 (6) Å^3^
*Z* = 4
Mo *K*α radiation
μ = 0.16 mm^−1^
*T* = 293 K
0.35 × 0.34 × 0.17 mm

#### Data collection

Bruker APEX DUO diffractometer
Absorption correction: multi-scan (*SADABS*; Sheldrick, 1996 ▶) *T*_min_ = 0.946, *T*_max_ = 0.973
69257 measured reflections
14918 independent reflections
10508 reflections with *I* > 2σ(*I*)
*R*_int_ = 0.030

#### Refinement

*R*[*F* ^2^ > 2σ(*F* ^2^)] = 0.045
*wR*(*F* ^2^) = 0.135
*S* = 1.01
14918 reflections
609 parameters
37 restraints
H-atom parameters constrained
Δρ_max_ = 0.27 e Å^−3^
Δρ_min_ = −0.22 e Å^−3^

### 

Data collection: *APEX2* (Bruker, 2010 ▶); cell refinement: *SAINT* (Bruker, 2010 ▶); data reduction: *SAINT*; program(s) used to solve structure: *SHELXS97* (Sheldrick, 2008 ▶); program(s) used to refine structure: *SHELXL97* (Sheldrick, 2008 ▶); molecular graphics: *X-SEED* (Barbour, 2001 ▶); software used to prepare material for publication: *publCIF* (Westrip, 2010 ▶).

## Supplementary Material

Crystal structure: contains datablock(s) global, I. DOI: 10.1107/S1600536811052731/hg5152sup1.cif

Structure factors: contains datablock(s) I. DOI: 10.1107/S1600536811052731/hg5152Isup2.hkl

Supplementary material file. DOI: 10.1107/S1600536811052731/hg5152Isup3.cml

Additional supplementary materials:  crystallographic information; 3D view; checkCIF report

## Figures and Tables

**Fig. 1.**
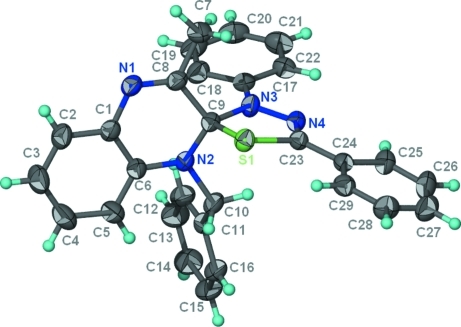
Thermal ellipsoid plot (Barbour, 2001) of one C29H24N4S molecule at the 50% probability level; hydrogen atoms are drawn as spheres of arbitrary radius.

**Fig. 2.**
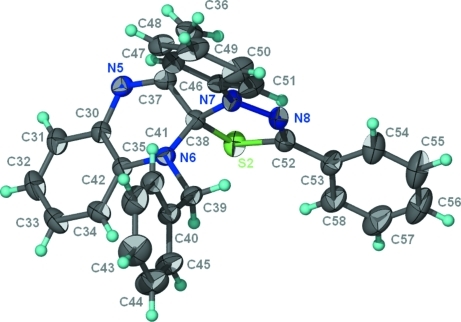
Thermal ellipsoid plot (Barbour, 2001) of second C29H24N4S molecule at the 50% probability level; hydrogen atoms are drawn as spheres of arbitrary radius. The disorder in the phenyl ring is now shown.

## supplementary crystallographic information

### Comment

A previous study reported 1-allyl-3-methyl-3',5'-diphenylspiro[quinoxaline-2(1*H*),2'(3'*H*)-[1,3,4]thiadiazole] (Anothane *et al.*, 2010). The allyl substitutent is replaced by a benzyl substituent in the present study. The asymmetric unit of C_29_H_24_N_4_S (Scheme I) consists of two independent molecules, one of which is disordered in the benzyl substituent. The quinoxaline and thiadiazole ring systems share a common C atom; their mean planes are aligned at 87.0 (1)° in one molecule (Fig.1 ) and at 84.1 (1)° in the other independent molecule (Fig. 2). The thiazole ring possesses two aromatic ring substituents and is nearly coplanar with these rings. The aromatic ring of the benzyl unit of one molecule is disordered over two positions in a 1:1 ratio.

### Experimental

To a solution of1-benzyl-3-methylquinoxaline-2-thione (1 g, 3.75 mmole) and diphenylnitrilimine (1.28 g, 5.55 mmole) in THF (20 mL), was added triethylamine (0.78 ml, 5.55 mmol). The mixture was heated under reflux for 24 hours. The precipitate was recovered by filtration and was separated by chromatography on silica gel (hexane/ethylAcetate: 9/1). Colorless crystals were isolated when solvent was allowed to evaporate.

### Refinement

Carbon-bound H-atoms were placed in calculated positions (C–H 0.93–0.97 Å) and were included in the refinement in the riding model approximation, with *U*(H) set to 1.2–1.5*U*(C).

One of the phenyl rings of the second independent molecule is disordered over two positions in 1:1 ratio. The ring was refined as a rigid hexagon of 1.39 Å sides. The temperature factors of the primed atoms were set to those of the unprimed ones, and all anisotropic temperature factors were restrained to be nearly isotropic. The pair of C_benzyl_–C_phenyl_ distances were restrained to within 0.01 Å of each other.

Omitted were (0 1 1), (0 1 1), (1 0 0) and (1 1 1).

### Crystal data

**Table:**

C_29_H_24_N_4_S	*Z* = 4
*M**_r_* = 460.58	*F*(000) = 968
Triclinic, *P*1	*D*_x_ = 1.284 Mg m^−^^3^
Hall symbol: -P 1	Mo *K*α radiation, λ = 0.71073 Å
*a* = 13.5441 (2) Å	Cell parameters from 9950 reflections
*b* = 14.8971 (2) Å	θ = 2.5–30.7°
*c* = 15.0149 (2) Å	µ = 0.16 mm^−^^1^
α = 66.431 (1)°	*T* = 293 K
β = 63.921 (1)°	Prism, colorless
γ = 65.275 (1)°	0.35 × 0.34 × 0.17 mm
*V* = 2383.45 (6) Å^3^	

### Data collection

**Table:**

Bruker APEX DUO diffractometer	14918 independent reflections
Radiation source: fine-focus sealed tube	10508 reflections with *I* > 2σ(*I*)
graphite	*R*_int_ = 0.030
ω scans	θ_max_ = 30.8°, θ_min_ = 1.6°
Absorption correction: multi-scan (*SADABS*; Sheldrick, 1996)	*h* = −19→19
*T*_min_ = 0.946, *T*_max_ = 0.973	*k* = −21→21
69257 measured reflections	*l* = −21→21

### Refinement

**Table:**

Refinement on *F*^2^	Primary atom site location: structure-invariant direct methods
Least-squares matrix: full	Secondary atom site location: difference Fourier map
*R*[*F*^2^ > 2σ(*F*^2^)] = 0.045	Hydrogen site location: inferred from neighbouring sites
*wR*(*F*^2^) = 0.135	H-atom parameters constrained
*S* = 1.01	*w* = 1/[σ^2^(*F*_o_^2^) + (0.0622*P*)^2^ + 0.5234*P*] where *P* = (*F*_o_^2^ + 2*F*_c_^2^)/3
14918 reflections	(Δ/σ)_max_ = 0.001
609 parameters	Δρ_max_ = 0.27 e Å^−^^3^
37 restraints	Δρ_min_ = −0.22 e Å^−^^3^

### Fractional atomic coordinates and isotropic or equivalent isotropic displacement parameters (Å^2^)

**Table:**

	*x*	*y*	*z*	*U*_iso_*/*U*_eq_	Occ. (<1)
S1	1.03210 (3)	0.16041 (3)	0.12571 (3)	0.04354 (9)	
S2	0.00042 (3)	0.90325 (3)	0.39048 (3)	0.04801 (10)	
N1	0.83510 (11)	0.22867 (9)	0.36228 (9)	0.0460 (3)	
N2	0.79303 (10)	0.23285 (9)	0.19279 (8)	0.0422 (2)	
N3	0.90581 (10)	0.34954 (9)	0.08824 (8)	0.0424 (2)	
N4	0.98330 (10)	0.33516 (8)	−0.00567 (8)	0.0392 (2)	
N5	0.19751 (10)	0.90389 (9)	0.14100 (8)	0.0417 (2)	
N6	0.20056 (9)	0.74996 (8)	0.32571 (8)	0.0363 (2)	
N7	0.01669 (9)	0.74698 (8)	0.34166 (8)	0.0382 (2)	
N8	−0.06977 (10)	0.74838 (9)	0.43523 (9)	0.0420 (2)	
C1	0.76113 (13)	0.17803 (10)	0.37494 (10)	0.0432 (3)	
C2	0.70620 (15)	0.12791 (12)	0.47415 (11)	0.0544 (4)	
H2	0.7206	0.1278	0.5293	0.065*	
C3	0.63089 (18)	0.07861 (14)	0.49172 (14)	0.0682 (5)	
H3	0.5945	0.0451	0.5583	0.082*	
C4	0.60992 (18)	0.07943 (15)	0.40958 (15)	0.0712 (5)	
H4	0.5587	0.0463	0.4214	0.085*	
C5	0.66327 (16)	0.12833 (13)	0.31002 (13)	0.0586 (4)	
H5	0.6481	0.1277	0.2556	0.070*	
C6	0.73990 (12)	0.17860 (10)	0.29116 (10)	0.0420 (3)	
C7	0.97184 (16)	0.32273 (14)	0.25839 (13)	0.0583 (4)	
H7A	0.9749	0.3136	0.3242	0.087*	
H7B	0.9432	0.3945	0.2276	0.087*	
H7C	1.0476	0.2954	0.2147	0.087*	
C8	0.89375 (13)	0.26797 (11)	0.27174 (10)	0.0433 (3)	
C9	0.89281 (12)	0.25828 (10)	0.17439 (9)	0.0382 (3)	
C10	0.78465 (13)	0.21995 (13)	0.10455 (11)	0.0492 (3)	
H10A	0.8047	0.1472	0.1124	0.059*	
H10B	0.8412	0.2465	0.0426	0.059*	
C11	0.66866 (12)	0.27151 (12)	0.08998 (10)	0.0444 (3)	
C12	0.59016 (14)	0.35441 (14)	0.12664 (13)	0.0560 (4)	
H12	0.6069	0.3788	0.1650	0.067*	
C13	0.48657 (16)	0.40219 (15)	0.10738 (15)	0.0651 (4)	
H13	0.4340	0.4577	0.1334	0.078*	
C14	0.46113 (17)	0.36769 (16)	0.04981 (15)	0.0681 (5)	
H14	0.3918	0.3999	0.0364	0.082*	
C15	0.53893 (17)	0.28544 (16)	0.01246 (14)	0.0666 (5)	
H15	0.5224	0.2622	−0.0269	0.080*	
C16	0.64163 (15)	0.23679 (14)	0.03277 (12)	0.0540 (4)	
H16	0.6931	0.1803	0.0079	0.065*	
C17	0.83014 (12)	0.44896 (10)	0.09100 (10)	0.0396 (3)	
C18	0.73607 (14)	0.46811 (13)	0.17849 (12)	0.0519 (3)	
H18	0.7201	0.4143	0.2362	0.062*	
C19	0.66687 (15)	0.56774 (14)	0.17868 (14)	0.0595 (4)	
H19	0.6044	0.5803	0.2370	0.071*	
C20	0.68894 (15)	0.64826 (13)	0.09420 (15)	0.0604 (4)	
H20	0.6424	0.7149	0.0955	0.073*	
C21	0.78075 (15)	0.62907 (12)	0.00762 (15)	0.0575 (4)	
H21	0.7954	0.6833	−0.0500	0.069*	
C22	0.85136 (13)	0.53058 (11)	0.00511 (12)	0.0464 (3)	
H22	0.9130	0.5188	−0.0539	0.056*	
C23	1.05153 (11)	0.24335 (10)	0.00069 (9)	0.0368 (3)	
C24	1.13657 (11)	0.20878 (10)	−0.09069 (10)	0.0378 (3)	
C25	1.14717 (13)	0.27713 (13)	−0.18831 (11)	0.0494 (3)	
H25	1.1034	0.3455	−0.1953	0.059*	
C26	1.22270 (15)	0.24296 (16)	−0.27435 (12)	0.0630 (4)	
H26	1.2289	0.2885	−0.3393	0.076*	
C27	1.28896 (15)	0.14228 (16)	−0.26508 (14)	0.0653 (5)	
H27	1.3395	0.1199	−0.3236	0.078*	
C28	1.28045 (14)	0.07476 (14)	−0.16929 (14)	0.0592 (4)	
H28	1.3261	0.0069	−0.1630	0.071*	
C29	1.20409 (12)	0.10748 (12)	−0.08214 (12)	0.0464 (3)	
H29	1.1980	0.0613	−0.0175	0.056*	
C30	0.28653 (11)	0.87214 (10)	0.18112 (10)	0.0397 (3)	
C31	0.37500 (13)	0.91778 (12)	0.12528 (13)	0.0528 (4)	
H31	0.3737	0.9673	0.0633	0.063*	
C32	0.46396 (14)	0.89049 (14)	0.16081 (15)	0.0618 (4)	
H32	0.5222	0.9219	0.1237	0.074*	
C33	0.46623 (14)	0.81610 (14)	0.25209 (15)	0.0592 (4)	
H33	0.5263	0.7977	0.2764	0.071*	
C34	0.38066 (13)	0.76849 (12)	0.30798 (13)	0.0493 (3)	
H34	0.3841	0.7179	0.3690	0.059*	
C35	0.28904 (11)	0.79598 (10)	0.27338 (10)	0.0373 (3)	
C36	0.01034 (15)	0.90929 (12)	0.15860 (13)	0.0549 (4)	
H36A	0.0288	0.9529	0.0899	0.082*	
H36B	−0.0579	0.9466	0.2022	0.082*	
H36C	−0.0025	0.8507	0.1584	0.082*	
C37	0.10810 (12)	0.87449 (9)	0.19810 (10)	0.0387 (3)	
C38	0.09188 (11)	0.81007 (9)	0.30892 (9)	0.0349 (2)	
C39	0.19346 (12)	0.68252 (11)	0.43048 (10)	0.0435 (3)	
H39A	0.1181	0.6718	0.4638	0.052*	0.50
H39B	0.1996	0.7177	0.4691	0.052*	0.50
H39C	0.1176	0.6729	0.4641	0.052*	0.50
H39D	0.2009	0.7173	0.4687	0.052*	0.50
C40	0.2843 (4)	0.5780 (3)	0.4369 (5)	0.0450 (3)	0.50
C41	0.3129 (5)	0.5183 (5)	0.3729 (4)	0.0530 (11)	0.50
H41	0.2802	0.5438	0.3213	0.064*	0.50
C42	0.3902 (6)	0.4206 (5)	0.3860 (6)	0.0691 (12)	0.50
H42	0.4093	0.3807	0.3431	0.083*	0.50
C43	0.4389 (5)	0.3825 (3)	0.4631 (7)	0.0853 (7)	0.50
H43	0.4907	0.3171	0.4718	0.102*	0.50
C44	0.4104 (5)	0.4421 (5)	0.5271 (5)	0.0808 (16)	0.50
H44	0.4430	0.4167	0.5787	0.097*	0.50
C45	0.3330 (4)	0.5399 (4)	0.5140 (5)	0.0592 (11)	0.50
H45	0.3139	0.5798	0.5569	0.071*	0.50
C40'	0.2816 (4)	0.5787 (3)	0.4372 (5)	0.0450 (3)	0.50
C41'	0.3403 (5)	0.5333 (5)	0.3561 (4)	0.0530 (11)	0.50
H41'	0.3291	0.5689	0.2927	0.064*	0.50
C42'	0.4159 (5)	0.4347 (5)	0.3696 (6)	0.0691 (12)	0.50
H42'	0.4552	0.4043	0.3152	0.083*	0.50
C43'	0.4326 (5)	0.3815 (3)	0.4642 (7)	0.0853 (7)	0.50
H43'	0.4831	0.3155	0.4732	0.102*	0.50
C44'	0.3738 (5)	0.4269 (5)	0.5454 (5)	0.0808 (16)	0.50
H44'	0.3851	0.3913	0.6087	0.097*	0.50
C45'	0.2983 (4)	0.5255 (4)	0.5319 (4)	0.0592 (11)	0.50
H45'	0.2590	0.5558	0.5862	0.071*	0.50
C46	0.05731 (12)	0.65744 (9)	0.30806 (10)	0.0373 (3)	
C47	0.13942 (13)	0.65090 (11)	0.21192 (11)	0.0441 (3)	
H47	0.1696	0.7053	0.1694	0.053*	
C48	0.17631 (14)	0.56422 (12)	0.17923 (12)	0.0508 (3)	
H48	0.2300	0.5613	0.1143	0.061*	
C49	0.13428 (18)	0.48211 (12)	0.24180 (13)	0.0617 (4)	
H49	0.1604	0.4232	0.2203	0.074*	
C50	0.05311 (19)	0.48863 (13)	0.33660 (13)	0.0657 (5)	
H50	0.0243	0.4334	0.3791	0.079*	
C51	0.01315 (15)	0.57533 (11)	0.37033 (11)	0.0510 (4)	
H51	−0.0429	0.5787	0.4342	0.061*	
C52	−0.08744 (12)	0.82234 (10)	0.46872 (11)	0.0431 (3)	
C53	−0.17437 (13)	0.83765 (11)	0.56788 (11)	0.0500 (3)	
C54	−0.26402 (16)	0.79480 (15)	0.61287 (15)	0.0689 (5)	
H54	−0.2698	0.7567	0.5801	0.083*	
C55	−0.3453 (2)	0.80904 (18)	0.70713 (17)	0.0888 (7)	
H55	−0.4058	0.7807	0.7372	0.107*	
C56	−0.3365 (2)	0.86476 (19)	0.75598 (17)	0.0921 (8)	
H56	−0.3907	0.8735	0.8193	0.111*	
C57	−0.2486 (2)	0.90736 (18)	0.71186 (16)	0.0849 (7)	
H57	−0.2432	0.9451	0.7453	0.102*	
C58	−0.16709 (17)	0.89472 (14)	0.61754 (14)	0.0643 (4)	
H58	−0.1077	0.9244	0.5876	0.077*	

### Atomic displacement parameters (Å^2^)

**Table:**

	*U*^11^	*U*^22^	*U*^33^	*U*^12^	*U*^13^	*U*^23^
S1	0.04818 (19)	0.03957 (17)	0.03708 (16)	−0.01054 (14)	−0.01453 (14)	−0.00653 (13)
S2	0.0507 (2)	0.03946 (18)	0.0531 (2)	−0.01480 (15)	−0.00821 (16)	−0.01978 (15)
N1	0.0593 (7)	0.0467 (6)	0.0350 (5)	−0.0181 (6)	−0.0160 (5)	−0.0105 (5)
N2	0.0467 (6)	0.0550 (7)	0.0311 (5)	−0.0223 (5)	−0.0101 (4)	−0.0124 (5)
N3	0.0486 (6)	0.0376 (6)	0.0320 (5)	−0.0109 (5)	−0.0085 (5)	−0.0079 (4)
N4	0.0421 (6)	0.0409 (6)	0.0326 (5)	−0.0144 (5)	−0.0095 (4)	−0.0086 (4)
N5	0.0503 (6)	0.0379 (6)	0.0376 (5)	−0.0180 (5)	−0.0151 (5)	−0.0047 (4)
N6	0.0397 (5)	0.0362 (5)	0.0328 (5)	−0.0117 (4)	−0.0140 (4)	−0.0059 (4)
N7	0.0415 (6)	0.0347 (5)	0.0390 (5)	−0.0169 (4)	−0.0087 (4)	−0.0094 (4)
N8	0.0395 (6)	0.0409 (6)	0.0402 (6)	−0.0136 (5)	−0.0080 (5)	−0.0093 (5)
C1	0.0519 (8)	0.0386 (7)	0.0360 (6)	−0.0124 (6)	−0.0125 (6)	−0.0099 (5)
C2	0.0698 (10)	0.0470 (8)	0.0361 (7)	−0.0185 (7)	−0.0136 (7)	−0.0041 (6)
C3	0.0809 (12)	0.0593 (10)	0.0493 (9)	−0.0349 (9)	−0.0102 (9)	0.0025 (8)
C4	0.0826 (13)	0.0679 (11)	0.0652 (11)	−0.0481 (10)	−0.0167 (10)	−0.0005 (9)
C5	0.0699 (11)	0.0620 (10)	0.0531 (9)	−0.0361 (9)	−0.0184 (8)	−0.0083 (7)
C6	0.0479 (7)	0.0397 (7)	0.0364 (6)	−0.0160 (6)	−0.0099 (5)	−0.0093 (5)
C7	0.0686 (10)	0.0716 (11)	0.0526 (9)	−0.0344 (9)	−0.0224 (8)	−0.0159 (8)
C8	0.0527 (8)	0.0463 (7)	0.0366 (6)	−0.0162 (6)	−0.0168 (6)	−0.0121 (5)
C9	0.0444 (7)	0.0397 (6)	0.0317 (5)	−0.0149 (5)	−0.0120 (5)	−0.0085 (5)
C10	0.0470 (8)	0.0714 (10)	0.0379 (7)	−0.0210 (7)	−0.0100 (6)	−0.0234 (7)
C11	0.0477 (7)	0.0595 (8)	0.0309 (6)	−0.0263 (7)	−0.0086 (5)	−0.0101 (6)
C12	0.0569 (9)	0.0674 (10)	0.0564 (9)	−0.0200 (8)	−0.0198 (7)	−0.0253 (8)
C13	0.0589 (10)	0.0691 (11)	0.0743 (11)	−0.0128 (8)	−0.0252 (9)	−0.0280 (9)
C14	0.0612 (11)	0.0815 (13)	0.0730 (12)	−0.0186 (9)	−0.0338 (9)	−0.0205 (10)
C15	0.0703 (11)	0.0897 (13)	0.0622 (10)	−0.0301 (10)	−0.0287 (9)	−0.0265 (10)
C16	0.0580 (9)	0.0695 (10)	0.0453 (8)	−0.0243 (8)	−0.0145 (7)	−0.0222 (7)
C17	0.0416 (7)	0.0397 (6)	0.0440 (7)	−0.0107 (5)	−0.0176 (5)	−0.0145 (5)
C18	0.0545 (9)	0.0536 (8)	0.0476 (8)	−0.0132 (7)	−0.0124 (7)	−0.0212 (7)
C19	0.0548 (9)	0.0656 (10)	0.0650 (10)	−0.0061 (8)	−0.0177 (8)	−0.0383 (9)
C20	0.0597 (10)	0.0470 (8)	0.0863 (12)	−0.0005 (7)	−0.0355 (9)	−0.0318 (9)
C21	0.0612 (10)	0.0408 (8)	0.0745 (11)	−0.0120 (7)	−0.0313 (9)	−0.0118 (7)
C22	0.0468 (8)	0.0419 (7)	0.0527 (8)	−0.0137 (6)	−0.0196 (6)	−0.0102 (6)
C23	0.0378 (6)	0.0404 (6)	0.0344 (6)	−0.0152 (5)	−0.0119 (5)	−0.0081 (5)
C24	0.0340 (6)	0.0462 (7)	0.0382 (6)	−0.0159 (5)	−0.0106 (5)	−0.0128 (5)
C25	0.0475 (8)	0.0547 (8)	0.0405 (7)	−0.0149 (7)	−0.0120 (6)	−0.0102 (6)
C26	0.0568 (10)	0.0858 (13)	0.0388 (7)	−0.0252 (9)	−0.0062 (7)	−0.0154 (8)
C27	0.0481 (9)	0.0910 (14)	0.0580 (10)	−0.0188 (9)	−0.0023 (7)	−0.0398 (10)
C28	0.0446 (8)	0.0620 (10)	0.0713 (11)	−0.0082 (7)	−0.0118 (7)	−0.0340 (9)
C29	0.0414 (7)	0.0483 (8)	0.0500 (8)	−0.0134 (6)	−0.0136 (6)	−0.0148 (6)
C30	0.0408 (7)	0.0393 (6)	0.0398 (6)	−0.0145 (5)	−0.0086 (5)	−0.0136 (5)
C31	0.0495 (8)	0.0525 (8)	0.0532 (8)	−0.0238 (7)	−0.0053 (7)	−0.0147 (7)
C32	0.0431 (8)	0.0649 (10)	0.0795 (12)	−0.0243 (7)	−0.0055 (8)	−0.0286 (9)
C33	0.0407 (8)	0.0648 (10)	0.0830 (12)	−0.0115 (7)	−0.0214 (8)	−0.0326 (9)
C34	0.0446 (8)	0.0521 (8)	0.0575 (8)	−0.0105 (6)	−0.0212 (7)	−0.0194 (7)
C35	0.0374 (6)	0.0378 (6)	0.0397 (6)	−0.0105 (5)	−0.0105 (5)	−0.0161 (5)
C36	0.0633 (10)	0.0492 (8)	0.0608 (9)	−0.0228 (7)	−0.0374 (8)	0.0029 (7)
C37	0.0480 (7)	0.0314 (6)	0.0395 (6)	−0.0138 (5)	−0.0192 (5)	−0.0044 (5)
C38	0.0379 (6)	0.0307 (6)	0.0369 (6)	−0.0118 (5)	−0.0119 (5)	−0.0082 (5)
C39	0.0476 (7)	0.0452 (7)	0.0324 (6)	−0.0100 (6)	−0.0151 (5)	−0.0070 (5)
C40	0.0494 (8)	0.0423 (7)	0.0388 (6)	−0.0112 (6)	−0.0173 (6)	−0.0056 (5)
C41	0.053 (3)	0.0546 (19)	0.0449 (18)	−0.0114 (15)	−0.0151 (18)	−0.0131 (14)
C42	0.070 (3)	0.0560 (18)	0.067 (2)	−0.0065 (16)	−0.015 (2)	−0.0238 (18)
C43	0.0932 (16)	0.0511 (10)	0.0841 (14)	0.0059 (10)	−0.0393 (13)	−0.0088 (10)
C44	0.095 (4)	0.062 (2)	0.068 (2)	−0.001 (2)	−0.047 (3)	−0.0011 (16)
C45	0.072 (3)	0.0533 (17)	0.046 (2)	−0.0124 (17)	−0.027 (2)	−0.0057 (15)
C40'	0.0494 (8)	0.0423 (7)	0.0388 (6)	−0.0112 (6)	−0.0173 (6)	−0.0056 (5)
C41'	0.053 (3)	0.0546 (19)	0.0449 (18)	−0.0114 (15)	−0.0151 (18)	−0.0131 (14)
C42'	0.070 (3)	0.0560 (18)	0.067 (2)	−0.0065 (16)	−0.015 (2)	−0.0238 (18)
C43'	0.0932 (16)	0.0511 (10)	0.0841 (14)	0.0059 (10)	−0.0393 (13)	−0.0088 (10)
C44'	0.095 (4)	0.062 (2)	0.068 (2)	−0.001 (2)	−0.047 (3)	−0.0011 (16)
C45'	0.072 (3)	0.0533 (17)	0.046 (2)	−0.0124 (17)	−0.027 (2)	−0.0057 (15)
C46	0.0468 (7)	0.0329 (6)	0.0382 (6)	−0.0134 (5)	−0.0196 (5)	−0.0069 (5)
C47	0.0515 (8)	0.0409 (7)	0.0431 (7)	−0.0173 (6)	−0.0150 (6)	−0.0108 (5)
C48	0.0623 (9)	0.0488 (8)	0.0474 (8)	−0.0124 (7)	−0.0212 (7)	−0.0190 (6)
C49	0.0979 (14)	0.0410 (8)	0.0591 (9)	−0.0187 (8)	−0.0360 (9)	−0.0158 (7)
C50	0.1113 (15)	0.0430 (8)	0.0541 (9)	−0.0393 (9)	−0.0283 (10)	−0.0048 (7)
C51	0.0756 (10)	0.0437 (7)	0.0401 (7)	−0.0295 (7)	−0.0189 (7)	−0.0044 (6)
C52	0.0410 (7)	0.0385 (7)	0.0426 (7)	−0.0088 (5)	−0.0107 (5)	−0.0103 (5)
C53	0.0476 (8)	0.0418 (7)	0.0430 (7)	−0.0038 (6)	−0.0091 (6)	−0.0108 (6)
C54	0.0635 (11)	0.0617 (11)	0.0612 (10)	−0.0210 (9)	0.0015 (8)	−0.0188 (8)
C55	0.0759 (14)	0.0783 (14)	0.0714 (13)	−0.0273 (11)	0.0134 (10)	−0.0182 (11)
C56	0.0928 (17)	0.0832 (15)	0.0558 (11)	−0.0134 (13)	0.0087 (11)	−0.0263 (11)
C57	0.0926 (16)	0.0863 (15)	0.0602 (11)	−0.0065 (12)	−0.0130 (11)	−0.0392 (11)
C58	0.0647 (10)	0.0639 (10)	0.0544 (9)	−0.0073 (8)	−0.0138 (8)	−0.0250 (8)

### Geometric parameters (Å, °)

**Table:**

S1—C23	1.7614 (13)	C27—H27	0.9300
S1—C9	1.8863 (14)	C28—C29	1.385 (2)
S2—C52	1.7571 (14)	C28—H28	0.9300
S2—C38	1.8837 (13)	C29—H29	0.9300
N1—C8	1.2748 (18)	C30—C31	1.3941 (19)
N1—C1	1.3997 (19)	C30—C35	1.3998 (18)
N2—C6	1.3964 (17)	C31—C32	1.372 (2)
N2—C9	1.4360 (17)	C31—H31	0.9300
N2—C10	1.4696 (17)	C32—C33	1.379 (3)
N3—N4	1.3741 (15)	C32—H32	0.9300
N3—C17	1.4098 (17)	C33—C34	1.381 (2)
N3—C9	1.4650 (16)	C33—H33	0.9300
N4—C23	1.2844 (17)	C34—C35	1.3961 (19)
N5—C37	1.2744 (17)	C34—H34	0.9300
N5—C30	1.4050 (18)	C36—C37	1.4979 (19)
N6—C35	1.3973 (16)	C36—H36A	0.9600
N6—C38	1.4405 (16)	C36—H36B	0.9600
N6—C39	1.4755 (16)	C36—H36C	0.9600
N7—N8	1.3792 (15)	C37—C38	1.5218 (17)
N7—C46	1.4200 (16)	C39—C40'	1.505 (4)
N7—C38	1.4674 (15)	C39—C40	1.526 (4)
N8—C52	1.2810 (18)	C39—H39A	0.9700
C1—C2	1.390 (2)	C39—H39B	0.9700
C1—C6	1.4045 (19)	C39—H39C	0.9700
C2—C3	1.373 (3)	C39—H39D	0.9700
C2—H2	0.9300	C40—C41	1.3900
C3—C4	1.376 (3)	C40—C45	1.3900
C3—H3	0.9300	C41—C42	1.3900
C4—C5	1.381 (2)	C41—H41	0.9300
C4—H4	0.9300	C42—C43	1.3900
C5—C6	1.393 (2)	C42—H42	0.9300
C5—H5	0.9300	C43—C44	1.3900
C7—C8	1.493 (2)	C43—H43	0.9300
C7—H7A	0.9600	C44—C45	1.3900
C7—H7B	0.9600	C44—H44	0.9300
C7—H7C	0.9600	C45—H45	0.9300
C8—C9	1.5311 (17)	C40'—C41'	1.3900
C10—C11	1.505 (2)	C40'—C45'	1.3900
C10—H10A	0.9700	C41'—C42'	1.3900
C10—H10B	0.9700	C41'—H41'	0.9300
C11—C12	1.374 (2)	C42'—C43'	1.3900
C11—C16	1.389 (2)	C42'—H42'	0.9300
C12—C13	1.384 (2)	C43'—C44'	1.3900
C12—H12	0.9300	C43'—H43'	0.9300
C13—C14	1.377 (3)	C44'—C45'	1.3900
C13—H13	0.9300	C44'—H44'	0.9300
C14—C15	1.370 (3)	C45'—H45'	0.9300
C14—H14	0.9300	C46—C51	1.3906 (18)
C15—C16	1.381 (2)	C46—C47	1.3930 (19)
C15—H15	0.9300	C47—C48	1.382 (2)
C16—H16	0.9300	C47—H47	0.9300
C17—C22	1.392 (2)	C48—C49	1.377 (2)
C17—C18	1.398 (2)	C48—H48	0.9300
C18—C19	1.385 (2)	C49—C50	1.375 (3)
C18—H18	0.9300	C49—H49	0.9300
C19—C20	1.374 (3)	C50—C51	1.384 (2)
C19—H19	0.9300	C50—H50	0.9300
C20—C21	1.377 (3)	C51—H51	0.9300
C20—H20	0.9300	C52—C53	1.4699 (19)
C21—C22	1.381 (2)	C53—C54	1.386 (2)
C21—H21	0.9300	C53—C58	1.389 (2)
C22—H22	0.9300	C54—C55	1.391 (3)
C23—C24	1.4675 (18)	C54—H54	0.9300
C24—C29	1.387 (2)	C55—C56	1.374 (4)
C24—C25	1.3963 (19)	C55—H55	0.9300
C25—C26	1.379 (2)	C56—C57	1.365 (4)
C25—H25	0.9300	C56—H56	0.9300
C26—C27	1.375 (3)	C57—C58	1.387 (3)
C26—H26	0.9300	C57—H57	0.9300
C27—C28	1.374 (3)	C58—H58	0.9300
			
C23—S1—C9	89.54 (6)	C32—C31—H31	119.6
C52—S2—C38	89.22 (6)	C30—C31—H31	119.6
C8—N1—C1	119.26 (12)	C31—C32—C33	119.40 (15)
C6—N2—C9	119.25 (11)	C31—C32—H32	120.3
C6—N2—C10	118.00 (11)	C33—C32—H32	120.3
C9—N2—C10	116.44 (10)	C32—C33—C34	121.01 (15)
N4—N3—C17	117.47 (10)	C32—C33—H33	119.5
N4—N3—C9	117.73 (10)	C34—C33—H33	119.5
C17—N3—C9	123.78 (11)	C33—C34—C35	120.31 (15)
C23—N4—N3	112.64 (10)	C33—C34—H34	119.8
C37—N5—C30	118.41 (11)	C35—C34—H34	119.8
C35—N6—C38	116.56 (10)	C34—C35—N6	123.09 (12)
C35—N6—C39	117.82 (11)	C34—C35—C30	118.51 (13)
C38—N6—C39	115.50 (10)	N6—C35—C30	118.40 (11)
N8—N7—C46	116.60 (10)	C37—C36—H36A	109.5
N8—N7—C38	116.70 (10)	C37—C36—H36B	109.5
C46—N7—C38	120.87 (10)	H36A—C36—H36B	109.5
C52—N8—N7	112.79 (11)	C37—C36—H36C	109.5
C2—C1—N1	118.06 (13)	H36A—C36—H36C	109.5
C2—C1—C6	119.99 (14)	H36B—C36—H36C	109.5
N1—C1—C6	121.93 (12)	N5—C37—C36	119.71 (12)
C3—C2—C1	120.80 (16)	N5—C37—C38	122.79 (12)
C3—C2—H2	119.6	C36—C37—C38	117.38 (12)
C1—C2—H2	119.6	N6—C38—N7	112.12 (10)
C2—C3—C4	119.17 (16)	N6—C38—C37	111.60 (10)
C2—C3—H3	120.4	N7—C38—C37	111.89 (10)
C4—C3—H3	120.4	N6—C38—S2	113.51 (8)
C3—C4—C5	121.48 (17)	N7—C38—S2	101.45 (8)
C3—C4—H4	119.3	C37—C38—S2	105.72 (8)
C5—C4—H4	119.3	N6—C39—C40'	115.8 (3)
C4—C5—C6	119.91 (16)	N6—C39—C40	115.6 (3)
C4—C5—H5	120.0	N6—C39—H39A	108.4
C6—C5—H5	120.0	C40—C39—H39A	108.4
C5—C6—N2	122.65 (13)	N6—C39—H39B	108.4
C5—C6—C1	118.65 (13)	C40—C39—H39B	108.4
N2—C6—C1	118.65 (12)	H39A—C39—H39B	107.5
C8—C7—H7A	109.5	N6—C39—H39C	108.3
C8—C7—H7B	109.5	C40'—C39—H39C	108.3
H7A—C7—H7B	109.5	C40—C39—H39C	109.2
C8—C7—H7C	109.5	N6—C39—H39D	108.3
H7A—C7—H7C	109.5	C40'—C39—H39D	108.3
H7B—C7—H7C	109.5	C40—C39—H39D	107.7
N1—C8—C7	119.18 (12)	H39C—C39—H39D	107.4
N1—C8—C9	123.41 (12)	C41—C40—C45	120.0
C7—C8—C9	117.35 (12)	C41—C40—C39	121.8 (5)
N2—C9—N3	111.50 (11)	C45—C40—C39	118.0 (5)
N2—C9—C8	112.01 (11)	C40—C41—C42	120.0
N3—C9—C8	112.22 (11)	C40—C41—H41	120.0
N2—C9—S1	113.06 (9)	C42—C41—H41	120.0
N3—C9—S1	100.67 (8)	C43—C42—C41	120.0
C8—C9—S1	106.80 (9)	C43—C42—H42	120.0
N2—C10—C11	115.34 (12)	C41—C42—H42	120.0
N2—C10—H10A	108.4	C42—C43—C44	120.0
C11—C10—H10A	108.4	C42—C43—H43	120.0
N2—C10—H10B	108.4	C44—C43—H43	120.0
C11—C10—H10B	108.4	C45—C44—C43	120.0
H10A—C10—H10B	107.5	C45—C44—H44	120.0
C12—C11—C16	118.34 (14)	C43—C44—H44	120.0
C12—C11—C10	123.07 (13)	C44—C45—C40	120.0
C16—C11—C10	118.52 (14)	C44—C45—H45	120.0
C11—C12—C13	121.00 (15)	C40—C45—H45	120.0
C11—C12—H12	119.5	C41'—C40'—C45'	120.0
C13—C12—H12	119.5	C41'—C40'—C39	123.1 (5)
C14—C13—C12	120.15 (17)	C45'—C40'—C39	116.8 (5)
C14—C13—H13	119.9	C42'—C41'—C40'	120.0
C12—C13—H13	119.9	C42'—C41'—H41'	120.0
C15—C14—C13	119.37 (17)	C40'—C41'—H41'	120.0
C15—C14—H14	120.3	C41'—C42'—C43'	120.0
C13—C14—H14	120.3	C41'—C42'—H42'	120.0
C14—C15—C16	120.58 (16)	C43'—C42'—H42'	120.0
C14—C15—H15	119.7	C42'—C43'—C44'	120.0
C16—C15—H15	119.7	C42'—C43'—H43'	120.0
C15—C16—C11	120.55 (16)	C44'—C43'—H43'	120.0
C15—C16—H16	119.7	C45'—C44'—C43'	120.0
C11—C16—H16	119.7	C45'—C44'—H44'	120.0
C22—C17—C18	119.13 (13)	C43'—C44'—H44'	120.0
C22—C17—N3	119.04 (12)	C44'—C45'—C40'	120.0
C18—C17—N3	121.81 (13)	C44'—C45'—H45'	120.0
C19—C18—C17	119.57 (15)	C40'—C45'—H45'	120.0
C19—C18—H18	120.2	C51—C46—C47	118.82 (12)
C17—C18—H18	120.2	C51—C46—N7	120.08 (12)
C20—C19—C18	121.12 (16)	C47—C46—N7	121.08 (11)
C20—C19—H19	119.4	C48—C47—C46	120.48 (13)
C18—C19—H19	119.4	C48—C47—H47	119.8
C19—C20—C21	119.21 (15)	C46—C47—H47	119.8
C19—C20—H20	120.4	C49—C48—C47	120.65 (15)
C21—C20—H20	120.4	C49—C48—H48	119.7
C20—C21—C22	121.01 (16)	C47—C48—H48	119.7
C20—C21—H21	119.5	C50—C49—C48	118.86 (14)
C22—C21—H21	119.5	C50—C49—H49	120.6
C21—C22—C17	119.94 (15)	C48—C49—H49	120.6
C21—C22—H22	120.0	C49—C50—C51	121.60 (15)
C17—C22—H22	120.0	C49—C50—H50	119.2
N4—C23—C24	122.09 (11)	C51—C50—H50	119.2
N4—C23—S1	115.57 (10)	C50—C51—C46	119.56 (15)
C24—C23—S1	122.28 (10)	C50—C51—H51	120.2
C29—C24—C25	119.00 (13)	C46—C51—H51	120.2
C29—C24—C23	120.89 (12)	N8—C52—C53	122.12 (13)
C25—C24—C23	120.06 (12)	N8—C52—S2	116.15 (10)
C26—C25—C24	119.86 (15)	C53—C52—S2	121.72 (11)
C26—C25—H25	120.1	C54—C53—C58	119.44 (16)
C24—C25—H25	120.1	C54—C53—C52	120.23 (15)
C27—C26—C25	120.69 (16)	C58—C53—C52	120.33 (15)
C27—C26—H26	119.7	C53—C54—C55	119.8 (2)
C25—C26—H26	119.7	C53—C54—H54	120.1
C28—C27—C26	119.90 (15)	C55—C54—H54	120.1
C28—C27—H27	120.0	C56—C55—C54	120.3 (2)
C26—C27—H27	120.0	C56—C55—H55	119.9
C27—C28—C29	120.17 (16)	C54—C55—H55	119.9
C27—C28—H28	119.9	C57—C56—C55	120.20 (19)
C29—C28—H28	119.9	C57—C56—H56	119.9
C28—C29—C24	120.36 (15)	C55—C56—H56	119.9
C28—C29—H29	119.8	C56—C57—C58	120.5 (2)
C24—C29—H29	119.8	C56—C57—H57	119.8
C31—C30—C35	120.04 (13)	C58—C57—H57	119.8
C31—C30—N5	117.99 (13)	C57—C58—C53	119.9 (2)
C35—C30—N5	121.97 (11)	C57—C58—H58	120.1
C32—C31—C30	120.71 (16)	C53—C58—H58	120.1
			
C17—N3—N4—C23	177.09 (11)	C32—C33—C34—C35	−0.8 (2)
C9—N3—N4—C23	−14.02 (16)	C33—C34—C35—N6	179.24 (13)
C46—N7—N8—C52	168.27 (12)	C33—C34—C35—C30	0.2 (2)
C38—N7—N8—C52	14.87 (16)	C38—N6—C35—C34	154.72 (12)
C8—N1—C1—C2	174.26 (14)	C39—N6—C35—C34	10.82 (18)
C8—N1—C1—C6	−7.0 (2)	C38—N6—C35—C30	−26.29 (16)
N1—C1—C2—C3	178.70 (16)	C39—N6—C35—C30	−170.18 (11)
C6—C1—C2—C3	0.0 (2)	C31—C30—C35—C34	0.84 (19)
C1—C2—C3—C4	−0.1 (3)	N5—C30—C35—C34	179.82 (12)
C2—C3—C4—C5	0.3 (3)	C31—C30—C35—N6	−178.21 (12)
C3—C4—C5—C6	−0.3 (3)	N5—C30—C35—N6	0.78 (18)
C4—C5—C6—N2	−177.38 (17)	C30—N5—C37—C36	178.37 (13)
C4—C5—C6—C1	0.1 (3)	C30—N5—C37—C38	2.43 (19)
C9—N2—C6—C5	−162.95 (14)	C35—N6—C38—N7	163.42 (10)
C10—N2—C6—C5	−11.8 (2)	C39—N6—C38—N7	−51.84 (14)
C9—N2—C6—C1	19.59 (19)	C35—N6—C38—C37	37.00 (14)
C10—N2—C6—C1	170.72 (13)	C39—N6—C38—C37	−178.26 (10)
C2—C1—C6—C5	0.0 (2)	C35—N6—C38—S2	−82.34 (11)
N1—C1—C6—C5	−178.63 (14)	C39—N6—C38—S2	62.39 (12)
C2—C1—C6—N2	177.62 (14)	N8—N7—C38—N6	101.30 (12)
N1—C1—C6—N2	−1.1 (2)	C46—N7—C38—N6	−50.89 (15)
C1—N1—C8—C7	179.48 (14)	N8—N7—C38—C37	−132.44 (11)
C1—N1—C8—C9	−3.3 (2)	C46—N7—C38—C37	75.37 (14)
C6—N2—C9—N3	−154.10 (12)	N8—N7—C38—S2	−20.15 (12)
C10—N2—C9—N3	54.32 (16)	C46—N7—C38—S2	−172.34 (9)
C6—N2—C9—C8	−27.40 (17)	N5—C37—C38—N6	−26.28 (17)
C10—N2—C9—C8	−178.98 (12)	C36—C37—C38—N6	157.69 (12)
C6—N2—C9—S1	93.32 (12)	N5—C37—C38—N7	−152.83 (12)
C10—N2—C9—S1	−58.26 (14)	C36—C37—C38—N7	31.14 (16)
N4—N3—C9—N2	−99.93 (13)	N5—C37—C38—S2	97.58 (13)
C17—N3—C9—N2	68.19 (16)	C36—C37—C38—S2	−78.45 (13)
N4—N3—C9—C8	133.49 (12)	C52—S2—C38—N6	−104.98 (9)
C17—N3—C9—C8	−58.39 (17)	C52—S2—C38—N7	15.49 (9)
N4—N3—C9—S1	20.24 (13)	C52—S2—C38—C37	132.37 (9)
C17—N3—C9—S1	−171.63 (11)	C35—N6—C39—C40'	−74.8 (3)
N1—C8—C9—N2	20.1 (2)	C38—N6—C39—C40'	140.9 (3)
C7—C8—C9—N2	−162.65 (13)	C35—N6—C39—C40	−73.8 (3)
N1—C8—C9—N3	146.37 (14)	C38—N6—C39—C40	141.9 (3)
C7—C8—C9—N3	−36.35 (18)	N6—C39—C40—C41	−44.8 (3)
N1—C8—C9—S1	−104.23 (15)	C40'—C39—C40—C41	61 (35)
C7—C8—C9—S1	73.06 (15)	N6—C39—C40—C45	139.6 (2)
C23—S1—C9—N2	102.97 (9)	C40'—C39—C40—C45	−114 (36)
C23—S1—C9—N3	−16.07 (9)	C45—C40—C41—C42	0.0
C23—S1—C9—C8	−133.39 (9)	C39—C40—C41—C42	−175.5 (4)
C6—N2—C10—C11	73.28 (18)	C40—C41—C42—C43	0.0
C9—N2—C10—C11	−134.78 (13)	C41—C42—C43—C44	0.0
N2—C10—C11—C12	24.2 (2)	C42—C43—C44—C45	0.0
N2—C10—C11—C16	−158.88 (14)	C43—C44—C45—C40	0.0
C16—C11—C12—C13	0.1 (2)	C41—C40—C45—C44	0.0
C10—C11—C12—C13	177.07 (16)	C39—C40—C45—C44	175.7 (4)
C11—C12—C13—C14	−0.7 (3)	N6—C39—C40'—C41'	−21.0 (4)
C12—C13—C14—C15	0.4 (3)	C40—C39—C40'—C41'	−95 (35)
C13—C14—C15—C16	0.5 (3)	N6—C39—C40'—C45'	162.5 (2)
C14—C15—C16—C11	−1.1 (3)	C40—C39—C40'—C45'	88 (36)
C12—C11—C16—C15	0.8 (2)	C45'—C40'—C41'—C42'	0.0
C10—C11—C16—C15	−176.31 (15)	C39—C40'—C41'—C42'	−176.4 (5)
N4—N3—C17—C22	−12.63 (18)	C40'—C41'—C42'—C43'	0.0
C9—N3—C17—C22	179.22 (12)	C41'—C42'—C43'—C44'	0.0
N4—N3—C17—C18	168.98 (13)	C42'—C43'—C44'—C45'	0.0
C9—N3—C17—C18	0.8 (2)	C43'—C44'—C45'—C40'	0.0
C22—C17—C18—C19	−0.9 (2)	C41'—C40'—C45'—C44'	0.0
N3—C17—C18—C19	177.49 (14)	C39—C40'—C45'—C44'	176.6 (4)
C17—C18—C19—C20	0.1 (2)	N8—N7—C46—C51	−2.40 (18)
C18—C19—C20—C21	0.7 (3)	C38—N7—C46—C51	149.81 (13)
C19—C20—C21—C22	−0.8 (3)	N8—N7—C46—C47	176.04 (12)
C20—C21—C22—C17	0.0 (2)	C38—N7—C46—C47	−31.75 (18)
C18—C17—C22—C21	0.9 (2)	C51—C46—C47—C48	−0.1 (2)
N3—C17—C22—C21	−177.58 (13)	N7—C46—C47—C48	−178.54 (13)
N3—N4—C23—C24	176.09 (11)	C46—C47—C48—C49	−1.3 (2)
N3—N4—C23—S1	−1.33 (15)	C47—C48—C49—C50	1.4 (3)
C9—S1—C23—N4	11.29 (11)	C48—C49—C50—C51	−0.1 (3)
C9—S1—C23—C24	−166.12 (11)	C49—C50—C51—C46	−1.2 (3)
N4—C23—C24—C29	−175.80 (12)	C47—C46—C51—C50	1.3 (2)
S1—C23—C24—C29	1.45 (17)	N7—C46—C51—C50	179.77 (15)
N4—C23—C24—C25	1.58 (19)	N7—N8—C52—C53	−178.98 (12)
S1—C23—C24—C25	178.82 (11)	N7—N8—C52—S2	−0.26 (16)
C29—C24—C25—C26	1.0 (2)	C38—S2—C52—N8	−9.95 (12)
C23—C24—C25—C26	−176.42 (14)	C38—S2—C52—C53	168.77 (12)
C24—C25—C26—C27	−0.8 (3)	N8—C52—C53—C54	−20.8 (2)
C25—C26—C27—C28	−0.2 (3)	S2—C52—C53—C54	160.53 (14)
C26—C27—C28—C29	1.0 (3)	N8—C52—C53—C58	158.86 (15)
C27—C28—C29—C24	−0.7 (2)	S2—C52—C53—C58	−19.8 (2)
C25—C24—C29—C28	−0.3 (2)	C58—C53—C54—C55	−0.2 (3)
C23—C24—C29—C28	177.15 (13)	C52—C53—C54—C55	179.46 (18)
C37—N5—C30—C31	−169.58 (13)	C53—C54—C55—C56	−0.4 (3)
C37—N5—C30—C35	11.42 (19)	C54—C55—C56—C57	0.6 (4)
C35—C30—C31—C32	−1.4 (2)	C55—C56—C57—C58	−0.1 (4)
N5—C30—C31—C32	179.57 (14)	C56—C57—C58—C53	−0.5 (3)
C30—C31—C32—C33	0.9 (3)	C54—C53—C58—C57	0.7 (3)
C31—C32—C33—C34	0.2 (3)	C52—C53—C58—C57	−178.99 (17)

## References

[bb1] Anothane, C. A., Bouhfid, R., Zouihri, H., Essassi, E. M. & Ng, S. W. (2010). *Acta Cryst.* E**66**, o3227. 10.1107/S1600536810046994 PMC3011801 21589517

[bb2] Barbour, L. J. (2001). *J. Supramol. Chem.* **1**, 189–191.

[bb3] Bruker (2010). *APEX2* and *SAINT* Bruker AXS Inc., Madison, Wisconsin, USA.

[bb4] Sheldrick, G. M. (1996). *SADABS* University of Göttingen, Germany.

[bb5] Sheldrick, G. M. (2008). *Acta Cryst.* A**64**, 112–122. 10.1107/S0108767307043930 18156677

[bb6] Westrip, S. P. (2010). *J. Appl. Cryst.* **43**, 920–925.

